# Impact of antigen specificity on CD4^+^ T cell activation in chronic HIV-1 infection

**DOI:** 10.1186/1471-2334-13-100

**Published:** 2013-02-25

**Authors:** Miranda Z Smith, Sonia Bastidas, Urs Karrer, Annette Oxenius

**Affiliations:** 1 Institute of Microbiology, Swiss Federal Institute of Technology (ETH) Zurich, Zurich, Switzerland; 2 Division of Infectious Diseases and Hospital Epidemiology, University Hospital, Zurich, Switzerland; 3 Current affiliation: Department of Infectious Diseases, Central Clinical School, Monash University; Centre for Virology, Burnet Institute, Melbourne, VIC, Australia; 4 Current affiliation: Kantonsspital Winterthur, Winterthur, Switzerland

**Keywords:** HIV, Immune activation, CD4^+^ T cells, Antigen persistence, CD38, HLA-DR

## Abstract

**Background:**

HIV infection induces chronic immune activation which is associated with accelerated disease progression; the causes of this activation, however, are incompletely understood. We investigated the activation status of CD4^+^ T cells specific for chronic herpes viruses and the non-persistent antigen tetanus toxoid (TT) in HIV positive and HIV negative donors to assess whether persistent infections contribute to chronic CD4^+^ T cell activation.

**Methods:**

Untreated HIV^+^ patients and healthy, aged matched controls were recruited and activation levels assessed and compared between cells specific for persistent and non-persistent antigens. Activation levels on antigen-specific CD4^+^ T cells were measured by intracellular cytokine staining following *in vitro* stimulation with various recall antigens (CMV, EBV, HSV, VZV and TT) in conjunction with cell surface phenotyping.

**Results:**

Activation levels of herpes virus-specific CD4^+^ T cell populations, assessed by co-expression of CD38 and HLA-DR, were significantly elevated in HIV^+^ individuals compared to normal controls and compared to TT-specific responses. In contrast, we found similar levels of activation of TT-specific CD4^+^ T cells in HIV^+^ and HIV^-^ donors.

**Conclusions:**

These results show a disparate distribution of immune activation within CD4^+^ T cell populations depending on their specificity and suggest that the elevated level of immune activation that characterizes chronic HIV infection may be influenced by the persistence of other antigens.

## Background

Chronic immune activation is a characteristic feature of HIV-1 infection contributing to CD4^+^ T cell loss and progression to AIDS and death [1-3]. In contrast, SIV infection of natural primate hosts does not cause chronic immune activation despite sustained systemic viral replication and thus disease progression and immunodeficiency are largely absent [4-6]. Persistent immune activation is characterized by elevated levels of serum cytokines and chemokines [7-9], B cell dysregulation (reviewed in [10]), interference with T cell homeostasis [11,12] and lymphocyte activation, reflected by expression of markers such as CD38 and HLA-DR [3,13], increased *in vivo* turnover [14,15] and enhanced spontaneous apoptosis [16]. Importantly, activation levels of both CD4^+^ and CD8^+^ T lymphocytes in HIV infection are strong predictors of disease progression [3,17] and viral control [13], however the causes of this activation are incompletely understood. Moreover, it is likely that the activation of CD4^+^ and CD8^+^ lymphocytes are mediated by distinct processes [18,19]. The mechanisms leading to the activation and depletion of CD4^+^ lymphocytes are of particular interest since their maintenance is critical in staving off the onset of AIDS.

It is clear that not only HIV-infected or HIV-specific T cells exhibit increased levels of activation and turn-over but also cells with other specificities [20]. Activated CD4^+^ T cells also represent ideal targets for productive HIV-infection, thereby fostering an activation-infection-cascade. Recently, increased levels of systemic lipopolysaccharide (LPS) and other bacterial products, possibly caused by microbial translocation in the intestine, have been implicated in the genesis of chronic immune activation in HIV-1 infection, mainly by innate immune mechanisms via pattern-recognition receptors (PRRs) and inflammatory cytokines [21,22]. Although PRR- and cytokine-mediated bystander activation independent of T cell receptor (TCR) triggering might provide direct stimulation for memory cells [23-26], a causal link between microbial translocation and TCR-independent immune activation remains to be shown.

We have recently shown a correlation between HIV-specific CD4^+^ T cell dynamics following HIV viral rebound, and CD4^+^ T cell dynamics specific for chronic herpes viruses such as cytomegalovirus and Epstein-Barr virus [27]. No correlation was demonstrated between the HIV-specific response and responses to non-persistent antigens. This may indicate that TCR-dependent signals provided by low levels of persistent antigen derived from herpesviruses may be involved in chronic T cell activation. In the current study, we extended these findings and compared the activation status of CD4^+^ T cells specific for persistent herpes viruses, and for the non-persistent antigen tetanus toxoid in patients with untreated HIV infection and in healthy controls. While in HIV-negative controls T cell specificity did not measurably influence the level of immune activation, we found significantly increased immune activation in herpesvirus- but not TT-specific CD4^+^ T cell populations of patients with untreated HIV-infection. These results underscore our hypothesis that persistent infections with herpesviruses substantially contribute to chronic immune activation in HIV-1 infection.

## Methods

### Study subjects

Donors with chronic untreated HIV clade B infection, detectable viral load (>5,000 copies/ml) and CD4^+^ counts >250/μl were recruited from the infectious disease outpatient clinic of the University Hospital of Zurich. Healthy, age-matched controls were likewise recruited. Patients were pre-screened by IFNγ ELISpot for antigen reactivity. The study protocol was approved by the hospital ethics committee and written informed consent was obtained according to the guidelines of the University Hospital Zurich. See Table 1 for detailed donor profiles.

**Table 1:** Donor profiles

**Donor ID**	**Gender**	**Age**	**HIV status**	**RNA copies/ml**	**CD4 cells/ul**	**CD4 (%)**
02RB	M	56	positive	160000	326	21
09GH	F	41	positive	64000	339	25
12VF	M	38	positive	39000	398	17
13OJ	M	42	positive	33000	426	31
19CT	M	46	positive	140000	323	29
21JH	M	40	positive	6800	293	28
23RB	M	24	positive	110000	598	37
24BZ	F	27	positive	34000	341	27
26PC	M	40	positive	37000	440	35
27BS	F	49	positive	26000	262	21
28JA	M	58	positive	24000	650	25
31PO	M	46	positive	130000	472	22
34GB	M	28	positive	8100	482	25
35DS	M	48	positive	6600	578	33
40TF	M	53	positive	11000	793	27
05KC	M	43	positive	35200	257	21
06MV	F	58	positive	73000	596	27
11CS	F	42	positive	19000	362	23
20YB	M	47	positive	16200	722	30
	**M 74%**	**Mean 43**				**Mean 27**
	**F 26%**	**SD 10**				**SD 5**
HD01	F	32	negative			56
HD03	M	28	negative			39
HD05	F	29	negative			36
HD09	F	31	negative			48
HD11	F	27	negative			31
HD12	M	45	negative			39
HD13	M	59	negative			39
HD14	F	53	negative			48
HD15	F	52	negative			50
HD16	F	55	negative			54
	**M 30%**	**Mean 41**				**Mean 44**
	**F 70%**	**SD 13**				**SD 8**

Demographics, HIV status and CD4 profiles of study participants. M = male, F = female, SD = standard deviation.

### Cell preparation and antigen stimulation

Approximately 80ml Ethylenediaminetetraacetic Acid (EDTA)-anticoagulated blood was drawn from each donor, and peripheral blood mononuclear cells (PBMC) were extracted by density centrifugation within 5 hours using Lymphocyte Separation Media (PAA Laboratories, Austria). The extracted PBMC were washed three times in 1× PBS (once at 300 × g and twice at 100 × g to remove platelets), then resuspended at 2 × 10^7^ cells/ml in RPMI supplemented with 10% foetal calf serum, 100 U/ml penicillin, 100 μg/ml streptomycin and 2 mM L-glutamine (all PAA) (RF-10) plus 20 μg/ml DNase (Roche, Germany).

Most samples were assayed with fresh PBMC, however for some samples, PBMC were cryopreserved. For analyzing frozen samples, autologous monocyte-derived dendritic cells (MDDCs) were prepared approximately 1 week prior to analysis. 10% MDDCs were added to the thawed PBMC for analysis.

Antigen stimulations were performed using 2 × 10^6^ cells in 96-well U-bottomed plates in a final volume of 200 μl. Stimulations were performed with HIV peptides (NIH AIDS Research & Reference Reagent Program, 5 μg/ml/peptide), cytomegalovirus (CMV) lysate (Virion, Germany; 50 g/ml), Epstein-Barr virus (EBV) lysate (Virusys, USA; 18.75 μg/ml), Herpes simplex virus-1 & -2 lysate (Virion, 18.75 μg/ml), Varicella Zoster virus (VZV) lysate (Virion, 2.5 μg/ml) and Tetanus Toxoid (Chiron, Germany; 31.1 μg/ml) in conjunction with 0.5 μl each anti-CD28 (Becton Dickinson, clone 28.2) and anti-CD49d (BD, clone 9F10). Cells were cultured at 37°C, 5% CO_2_ for 2 hours, then 10 μg/ml Brefeldin A was added and cells cultured for a further 4 hours at 37°C. Samples were then held at 4°C overnight.

### Intracellular cytokine staining and T cell phenotyping

Cells were surface stained with anti-CD3 Pacific Blue (clone UCHT1), anti-CD45RA PE (clone HI100), anti-CD4 PerCP (clone SK3), anti-CD38 APC (clone HIT2) and anti-HLA-DR APC-H7 (clone L243), followed by permeabilisation and intracellular staining with anti-IFNγ PE-Cy7 (clone B27) (all from BD) and anti-IL-2 FITC (BioLegend, clone MQ1-17H12). Additional phenotyping was perfomed using anti-PD-1 PE-Cy7 (BioLegend, clone EH12.2H7) and anti-β7 integrin (eBioscience, clone FIB504). Samples were acquired on a three-laser, 9- parameter LSR II Flow cytometer (BD), and analysis performed using FlowJo v7.5.5 (Treestar, USA). Doublets were excluded using side- and forward-scatter height and width parameters, and CD4^+^CD45RA^-^ and CD4^+^CD45RA^+^ lymphocytes analysed for cytokine production and activation. Fluorescence minus one controls were used to set gates for HLA-DR, CD38, PD-1 and β7 integrin [28], and DMSO or co-stimulation-only samples used to set gates for cytokine production. Responses greater than or equal to two times background were considered positive.

### Statistical analysis

Results were analysed and graphed using Prism v5.04 (GraphPad Software, USA). Unpaired, 2-tailed t tests and Mann–Whitney tests were used to calculate significance. Correlation analysis used 2-tailed Pearson correlation.

## Results

### HIV^+^ donors show elevated levels of CD4^+^ T cell activation

**Figure 1:**
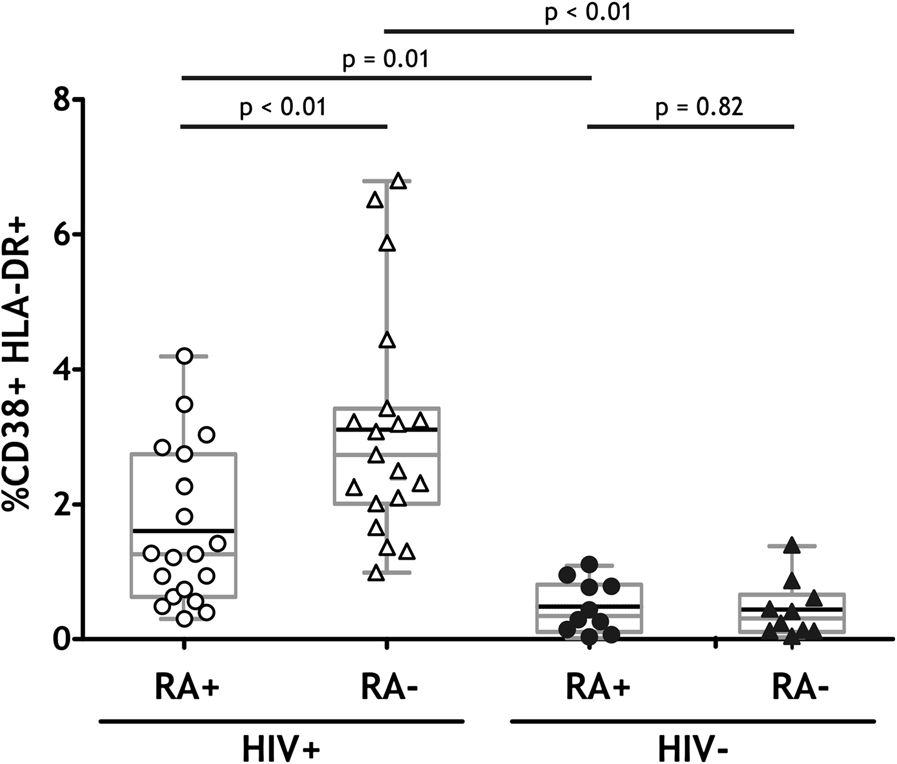
**Comparison of activation levels in CD4**^**+ **^**subsets between HIV**^**+ **^**and HIV- donors.** PBMC samples from 19 HIV^+^ and 10 HIV^-^ donors were characterized by flow cytometry. The CD3^+^CD4^+^CD45RA^+^ and CD3^+^CD4^+^CD45RA^-^ populations were assessed for expression of both CD38 and HLA-DR. Activation levels of the CD4^+^ subsets from HIV^+^ donors (open symbols), and HIV^-^ donors (filled symbols) are shown. Box and whisker plots (in grey) extend from the 5^th^ to the 95^th^ percentile, and the black line depicts the mean. Unpaired 2-tailed t-tests were conducted to compare activation levels between HIV^+^ and HIV^-^ populations, and Mann–Whitney tests used to compare levels within HIV^+^ or HIV^-^ populations.

Overall activation levels, evaluated by co-expression of CD38 and HLA-DR, were compared on CD4^+^CD45RA^+^ (naïve and terminally differentiated) and CD4^+^CD45RA^-^ (antigen experienced) subsets in HIV^+^ and HIV- donors (Figure 1). In HIV^-^ donors, both populations had low levels of activation (CD4^+^CD45RA^+^: mean 0.47% CD38^+^HLA-DR^+^; CD4^+^CD45RA^-^: mean 0.42% CD38^+^HLA-DR^+^). As expected, the activation levels in the HIV^+^ donors were significantly higher for both populations (CD4^+^CD45RA^+^: mean 1.50%, p < 0.01; CD4^+^CD45RA-: mean 2.98%, p < 0. 01). The activation level of the CD4^+^CD45RA- population was significantly higher than the CD4^+^CD45RA^+^ population (p < 0.01), suggesting that antigen encounter may influence activation levels in HIV^+^ individuals. While CD38 and HLA-DR expression were significantly higher in HIV^+^ than HIV^-^ individuals, only a minority of CD4^+^ T cells displayed an activated phenotype.

### Activated CD4 T cells in HIV^+^ and HIV^-^ donors express PD-1 and are β7 integrin negative

**Figure 2:**
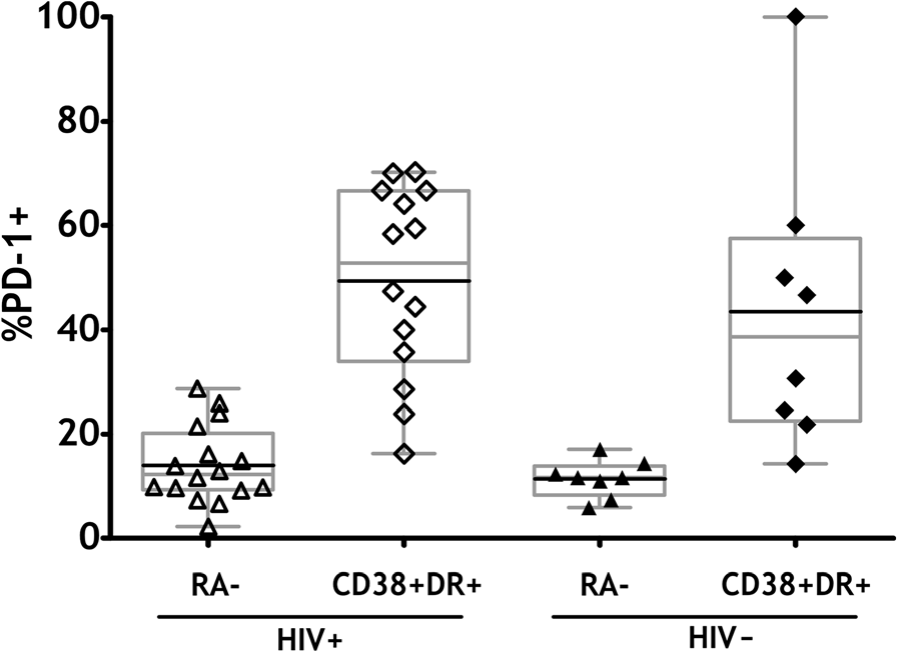
**PD-1 expression within memory and activated memory subsets.** PBMC samples from a subset of HIV^+^ and HIV^-^ donors were assessed for PD-1 expression by flow cytometry. PD-1 expression in total memory (CD45RA^-^) and activated (CD38^+^HLA^-^DR^+^) CD4^+^ T cells in HIV^+^ donors (open symbols), and HIV- donors (filled symbols) are depicted. Box and whisker plots (in grey) extend from the 5^th^ to the 95^th^ percentile, and the black line depicts the mean. Unpaired 2-tailed t-tests were conducted to compare PD-1 levels between HIV^+^ and HIV^-^ populations, and Mann–Whitney tests used to compare levels within HIV^+^ or HIV^-^ populations.

To address whether CD4^+^ T cells with an activated phenotype defined by HLA-DR and CD38 co-expression could be distinguished between HIV^+^ and HIV^-^ individuals, we performed co-staining for PD-1 and integrin β7. PD-1 is an activation marker associated with T cell exhaustion in chronic viral infections [6,29] and is expressed on a large proportion of T cells specific for viruses with a high level of persistence such as HIV, HBV and HCV [14-16]. β7 is an integrin which marks T cells with specific gut homing properties [17]. We chose these two additional markers to evaluate the activation status of the cells using an independent activation marker (PD-1) and to assess whether CD4 T cell activation in HIV patients might be disproportionally evident on cells with gut-homing potential (α4β7), possibly as a consequence of increased priming of microbiota-specific T cells in the setting of increased gut permeability. We did not find integrin β7 expression on HLA-DR and CD38 co-expressing cells from either HIV^+^ or HIV^-^ donors (data not shown, Additional file 1: Figure S1). PD-1 expression was elevated on CD38 and HLA-DR expressing cells compared to the bulk CD4^+^CD45RA- population in both HIV^+^ and HIV^-^ donors (Figure 2).

### Magnitude and functionality of antigen-specific CD4^+^ T cell responses in HIV^+^ and HIV^-^ individuals

**Figure 3:**
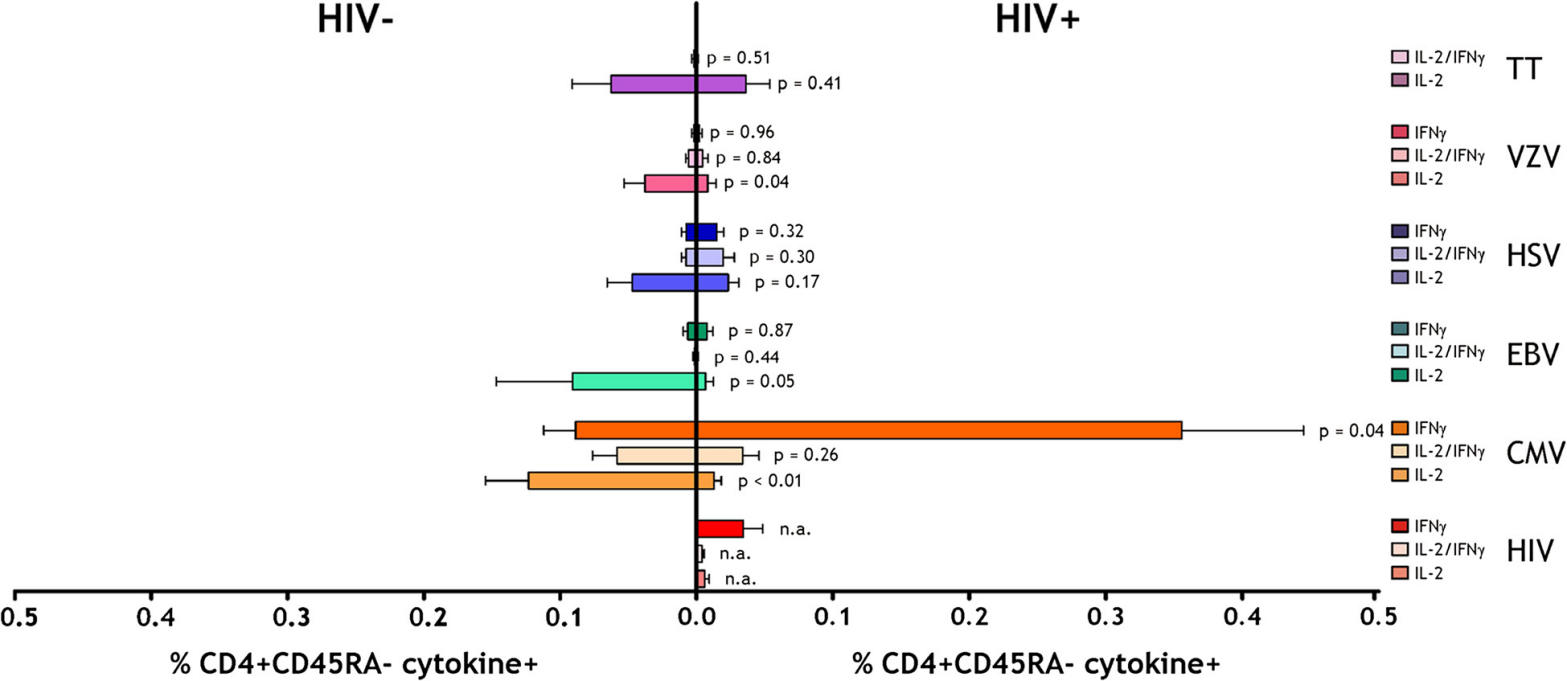
**Antigen-specific cytokine responses detected to persistent herpes viruses and tetanus toxoid.** Following a six hour stimulation, PBMCs were assessed by intracellular cytokine staining for responses to HIV, herpes viruses CMV, EBV, HSV & VZV, and the bacterial antigen tetanus toxoid. Responses were categorized according to the combination of cytokines produced (either IL-2 or IFNγ alone, or both together). Cytokine responses for each antigen are shown with HIV^-^ donors on the left and HIV^+^ donors on the right. Unpaired 2-tailed t-tests were used to compare responses between HIV^+^ and HIV^-^ donors.

Following *in vitro* stimulation, we detected IL-2 and IFNγ producing CD4^+^ T cell responses to a range of antigens including persistent herpes viruses (CMV, EBV, HSV, VZV) and a non-persistent bacterial vaccine antigen (TT) (Figure 3). Responses to the viral antigens included IL-2, IFNγ and dual-expressing cells, whereas only IL-2 production was detected in response to TT. While IL-2 responses to TT did not differ in HIV^+^ and HIV^-^ donors (p = 0.41), frequencies of herpes-virus specific IL-2 producing cells were generally higher in HIV^-^ donors, reaching statistical significance for CMV (p < 0.01), EBV (p = 0.05) and VZV (p = 0.04) (Figure 3). The same trend was seen for HSV, although the difference did not reach significance. In contrast, frequencies of IFNγ-producing cells largely did not differ between HIV^+^ and HIV^-^ donors with only CMV-specific IFNγ responses higher in HIV^+^ donors (p = 0.04) (EBV p = 0.87, HSV p = 0.32, VZV p = 0.96, TT = not detected in either group).

### Elevated activation levels of herpes-virus specific responses in HIV^+^ donors

Next, we studied the extent of CD38 and HLA-DR co-expression on the cytokine producing T cell populations. Before doing so, we had to rule out that the short-term antigen stimulation itself would induce any changes in CD38 or HLA-DR expression. We therefore calculated the fold change in CD38/HLA-DR co-expression of SEB stimulated cultures compared to control cultures and plotted these changes against the background level of CD38 and HLA-DR expression. We found no correlation (Additional file 2: Figure S2), and therefore concluded that any change induced by the assay itself was minimal and affected all samples to an equal extent.

**Figure 4:**
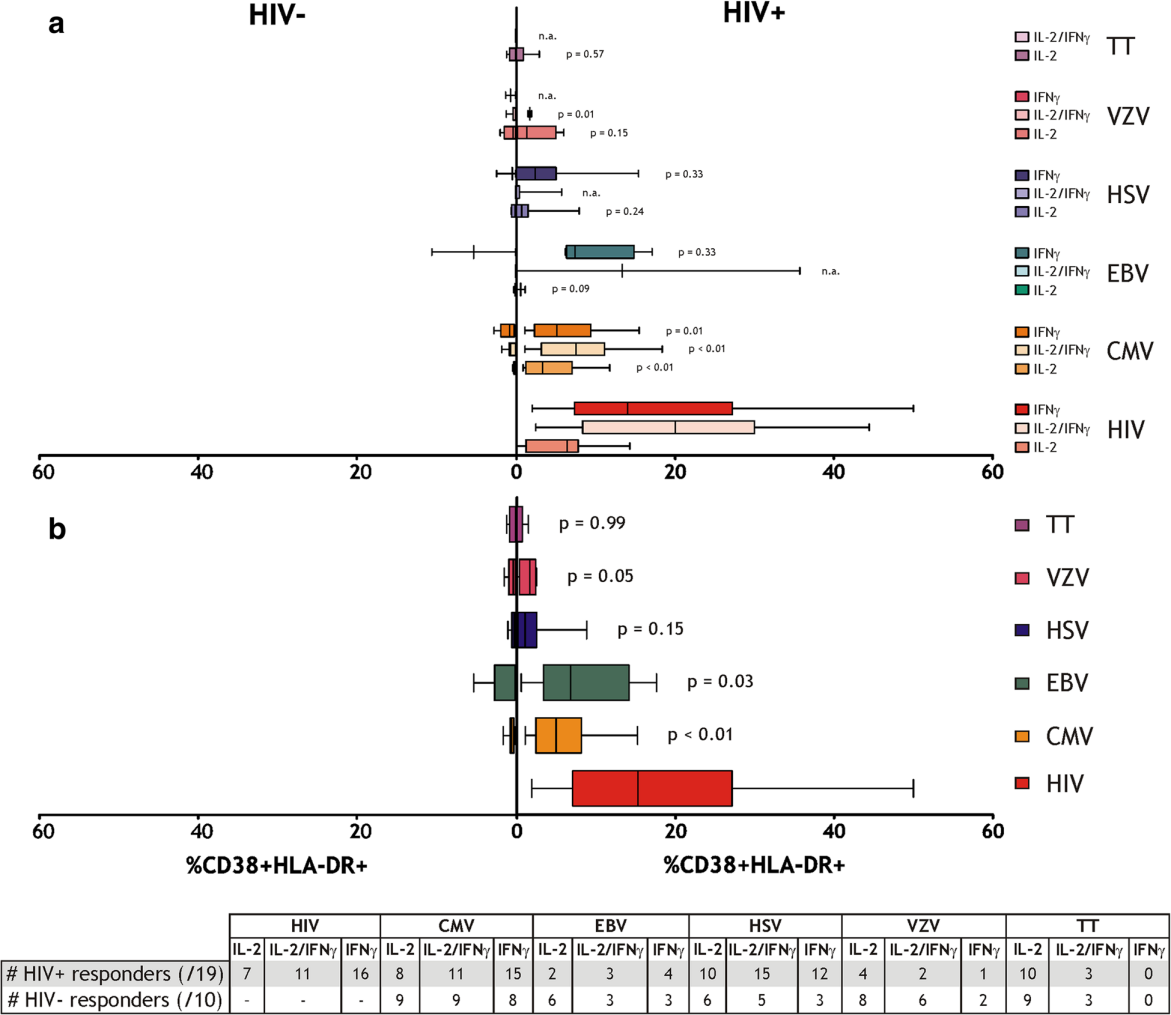
**Comparison of activation levels of antigen-specific populations between HIV**^**+ **^**and HIV**^**- **^**donors.** Antigen-specific responses (depicted in Figure 3) were assessed for expression of the activation markers CD38 and HLA-DR. (**a**) Activation levels for each combination of cytokines are depicted for the five persistent viral antigens and one non-persistent bacterial antigen. (**b**) Average activation levels for all cells responding to each antigen. The table below depicts the number of donors responding to each antigen by the three combinations of cytokines. Unpaired 2-tailed t-tests compared activation levels between HIV^+^ and HIV^-^ donors.

When assessing CD38 and HLA-DR expression on cytokine producing cells in HIV^+^ and HIV^-^ responders, we observed striking differences, with very few activated cells detected in HIV^-^ responders. Moreover, activation levels were similar in herpesvirus- and TT-specific CD4^+^ T cell populations and thus independent of antigen specificity or persistence in HIV^-^ controls (Figure 4b). In contrast, the levels of activation seen in HIV^+^ donors were significantly higher for CMV, EBV and VZV-specific responses and in some cases exceeded 20% of cytokine producing cells. In Figure 4a, the activation levels of single IL-2 or IFNγ-producing cells are shown along with the levels for cells producing both cytokines in response to all six antigens. In HIV^+^ donors, HIV-specific cells producing IFNγ show higher activation levels than cells producing IL-2 only, however activation levels did not significantly differ between response profiles for the other antigens. When we compared the activation levels of the responses between HIV^+^ and HIV^-^ donors, the CMV-specific responses all displayed significantly higher levels of activation in the HIV^+^ donors, however low numbers of responders by each cytokine combination meant that activation levels for the other antigens did not reach significance.

Remarkably, the TT-specific responses showed similar low levels of activation in both HIV^+^ and HIV^-^ donors. Lastly, the mean T cell activation in HIV^+^ individuals was highest for HIV-specific CD4^+^ T cells and was significantly higher than the activation levels of CMV, HSV and TT-specific CD4^+^ T cells (all p < 0.01).

## Discussion

We have studied immune activation levels and cytokine responses to a panel of persistent herpesviruses (CMV, EBV, HSV and VZV) and a non-persistent vaccine antigen (TT) in a cohort of patients with untreated chronic HIV-1 infection and compared them to responses in HIV-negative controls. While activated CD4^+^ T cells displayed elevated levels of the inhibitory marker PD-1 in both HIV^+^ and HIV^-^ individuals, the overall levels of activated CD4^+^ T cells were elevated only in the setting of HIV infection, as shown to be characteristic of chronic HIV infection [3,13]. In HIV-negative controls, frequencies of activated CD4^+^ T cells were very low and independent of antigen specificity or persistence. In HIV^+^ individuals, HIV-specific CD4^+^ T cells displayed the highest level of activation, followed by EBV, CMV, HSV, VZV and TT-specific cells. This ‘hierarchy’ may correlate with the level of persistence or the likelihood of reactivation/re-encounter of the respective virus/antigen, although this has not been formally addressed but may be inferred from the differentiation profile of CD8^+^ T cells specific for these viruses/antigens [30,31]. Moreover, responses to the persistent herpesviruses all demonstrated higher activation levels in HIV^+^ individuals than HIV^-^, a phenomenon not observed for responses to the non-persistent antigen TT. These results are compatible with our theory that persistent antigens contribute to chronic immune activation in HIV-positive individuals. An alternative explanation for our findings may be that some of our HIV^+^ patients may have been sufficiently immunodeficient for relevant herpesvirus reactivation. However, we did not find clinical evidence for viral reactivations and there was no correlation between total CD4-counts (or percentages) and the level of immune activation.

PD-1 expression has been found to be elevated in HIV infection [14] and is linked to HIV disease stage [32]. We found higher percentages of PD-1 expressing cells in our HIV^+^ donors, however the level of PD-1 expression on CD38^+^HLA-DR^+^ cells was similar in both HIV^+^ and HIV^-^ donors, suggesting that CD38^+^HLA-DR^+^ cells may share features in spite of HIV status. Recently, PD-1 expression on CD38^+^HLA-DR^+^ T cells has led to the definition of a distinct subset of CD4^+^ T cells called deregulated effector memory (DEM) cells [33], however the authors of this study did not evaluate the percentage of PD-1 expression on the CD38^+^HLA-DR^+^ cells in their HIV^+^ and HIV^-^ cohorts. A study of the gene expression profiles of activated cells in HIV^+^ and HIV^-^ donors showed significant differences in activated cells in HIV-infected and HIV negative donors [34], with activated cells from HIV^+^ donors expressing many more cell cycle-associated and interferon-stimulated genes than activated cells from HIV negative donors [34].

We detected both IFNγ and IL-2 responses to our panel of antigens. HIV- and CMV-specific responses were dominated by IFNγ producing cells; a mixture of IFNγ and IL-2 was found for EBV, HSV and VZV specific responses and the TT response was almost completely IL-2 mediated. The detection of IFNγ, and not IL-2, in response to HIV and CMV is consistent with the observed hierarchy of cytokine response loss in CD8^+^ T cells during chronic HIV infection [35]. Studies of the maturation status of antigen-specific CD4^+^ T cells have shown that CMV-specific responses are more mature than both HIV and TT-specific responses [19,23], and that CMV responses preferentially involve IFNγ production, while TT responses are mainly IL-2 mediated [23]. The lack of IL-2 responses to CMV also fits with recent data showing that CMV-specific CD4^+^ T cells produce little IL-2 but high levels of the CCR5 chemokine MIP-1β [36]. Geldmacher and colleagues showed that IL-2 producing cells are more vulnerable to HIV infection *in vivo* and are therefore deleted. Our data could be interpreted through this paradigm, with maintenance of IFNγ-producing cells specific for herpesviruses which are poor IL-2 producers, and the TT-specific cells maintained to similar levels in HIV^+^ and HIV^-^ individuals potentially because although they produce IL-2, they are not activated and are therefore poor targets for HIV replication.

The higher levels of activation seen in herpes-virus specific cells, in contrast to the low activation levels seen on TT-specific cells, suggests that these cells may be more susceptible to bystander activation. Bystander activation is a phenomenon primarily described for CD8^+^ T cells [24,37], but which has also been clearly demonstrated to occur in CD4^+^ T cells [26,38]. Bystander activation occurs via the actions of cytokines, TLR ligands and/or indirect mechanisms leading to a partial activation of T cells of varying specificities. However, it has yet to be demonstrated in humans that bystander activation alone is sufficient for the simultaneous up-regulation of HLA-DR and CD38 in the absence of any TCR-mediated signals. A recent study from our group demonstrated that the dynamics of responses to persistent herpesviruses, but not non-persisting antigens, closely mirrored the HIV-specific response on HIV viral rebound [27]. This observation was linked to dendritic cell activation and improved antigen presentation, and was dependent on the presence of a low level of cognate antigen. Similar observations have been made with CD8^+^ T cell responses, with simultaneous fluctuations of CMV and EBV-specific cells noted in persistent infection [39]. Moreover, primary HIV infection has been shown to cause low-level activation of CD8^+^ T cells specific for CMV and EBV (and influenza, although this is not a persistent antigen) [40,41] and treating HIV-infected patients with an anti-herpesvirus drug reduced CD8^+^ T cell activation [42]. A study of patients with a variety of acute viral infections demonstrated that all acute infections (including dengue, hepatitis B and adenovirus) activated herpesvirus-specific CD8^+^ T cells but not influenza-specific CD8^+^ T cells [29]. In this case, the authors suggest that the action of IL-15 contributes to this selective activation of herpesvirus-specific cells.

There are some limitations to the study presented here. Due to current treatment guidelines most patients are treated early with antiretroviral therapy, limiting the patients eligible for our study. In addition, a substantial number of potential participants were excluded after pre-screening by ELISpot since we were unable to detect robust CD4^+^ T cell responses against the antigens of interest. We analyzed IFNγ and IL-2 production, however CD4^+^ T cells can also produce significant quantities of TNFα, MIP-1β and IL-17, which could be assessed to give a broader representation of CD4 responses. Finally, to corroborate our findings, cells specific for non-persistent antigens other than TT, such as influenza and adenovirus, could be studied. We attempted to measure frequencies of adenovirus-specific CD4^+^ T cell responses in a number of HIV^+^ individuals in our cohort. In almost all patients, however, these responses were below the detection limit.

CD4^+^ T cells co-expressing HLA-DR, CD38 and PD-1 have previously been characterized as DEM cells in chronically infected Ugandans [33]. More detailed analysis of the DEM cells demonstrated that HIV- and CMV-specific responses were enriched amongst them, and that they exhibited a diverse Vβ repertoire, suggesting that the population is comprised of cells of diverse antigen specificities [33]. This study, along with the data presented here, suggests that the CD4^+^CD38^+^HLA-DR^+^ population could be considered a distinct population, sharing an elevated level of PD-1 expression, lack of integrin β7, and as recently demonstrated, an elevated expression of the HIV co-receptor CCR5 [43]. This CD4^+^CD38^+^HLA-DR^+^ population may represent cells specific for a variety of persistent antigens receiving some level of TCR-dependent triggering which are preferentially activated in the presence of inflammatory cytokines and TLR ligands, both hallmarks of chronic HIV infection.

## Conclusions

The data presented in this paper further characterize the nature of activated CD4^+^ T cells in untreated HIV infection. We demonstrate the influence of antigen exposure on susceptibility to activation, and show that CD4^+^ T cells specific for persistent antigens are more activated than those specific for non-persisting antigens. These observations suggest a mechanism contributing to the generalized immune activation seen in HIV infection, which is an important step towards developing strategies to limit the pathological activation linked to HIV disease progression.

## Abbreviations

HIV: Human immunodeficiency virus type 1; TT: Tetanus toxoid; CMV: Cytomegalovirus; HSV: Herpes simplex virus; EBV: Epstein-Barr virus; VZV: Varicella zoster virus; TCR: T cell receptor; PRR: Pattern recognition receptor; TLR: Toll-like receptor; DEM: Deregulated effector memory.

## Competing interests

The authors declare that they have no competing interests.

## Authors’ contributions

AO and UK designed the study. MS and SB performed the experiments. MS analysed all data. MS and AO wrote the manuscript. All authors read and approved the final manuscript.

## Pre-publication history

The pre-publication history for this paper can be accessed here:

http://www.biomedcentral.com/1471-2334/13/100/prepub

## Supplementary Material

Additional file 1: Figure S1 Gating for PD-1 and β7 expression. Fluorescence minus one (FMO) gating shown on CD4+ lymphocytes. The left plot shows the FMO control for PD-1 PE-Cy7, the middle plot shows the FMO control for β7 FITC and the right plot shows the resulting gates on a sample containing all antibodies. All control samples were derived from the same donor and analysed together. Click here for file

Additional file 2: Figure S2 Intracellular cytokine staining procedure has minimal effect on activation levels. The expression of CD38 and HLA-DR induced by the intracellular cytokine assay was assessed by comparing activation levels in the DMSO (negative) and SEB (positive) controls. Here the fold change demonstrated between DMSO and SEB is graphed against the percentage of activated CD4^+^ T cells in the DMSO control. There is no significant correlation (r = −0.18, p = 0.33). Donors with higher baseline levels of activation did not show any greater increase in activation through the assay than those with lower baseline levels of activation. Overall, the activation induced by the assay was no more than one times the background level. Click here for file
