# Supplementary material for: Impact of antigen specificity on CD4+ T cell activation in chronic HIV-1 infection
Source: BMC Infect Dis. 2013 Feb 25;13:100. doi: 10.1186/1471-2334-13-100 (PMC3598342; doi:10.1186/1471-2334-13-100)
Supplement: Additional file 1: Figure S1 — Gating for PD-1 and β7 expression. Fluorescence minus one (FMO) gating shown on CD4+ lymphocytes. The left plot shows the FMO control for PD-1 PE-Cy7, the middle plot shows the FMO control for β7 FITC and the right plot shows the resulting gates on a sample containing all antibodies. All control samples were derived from the same donor and analysed together. [file 1471-2334-13-100-S1.ppt]

## Slide 1
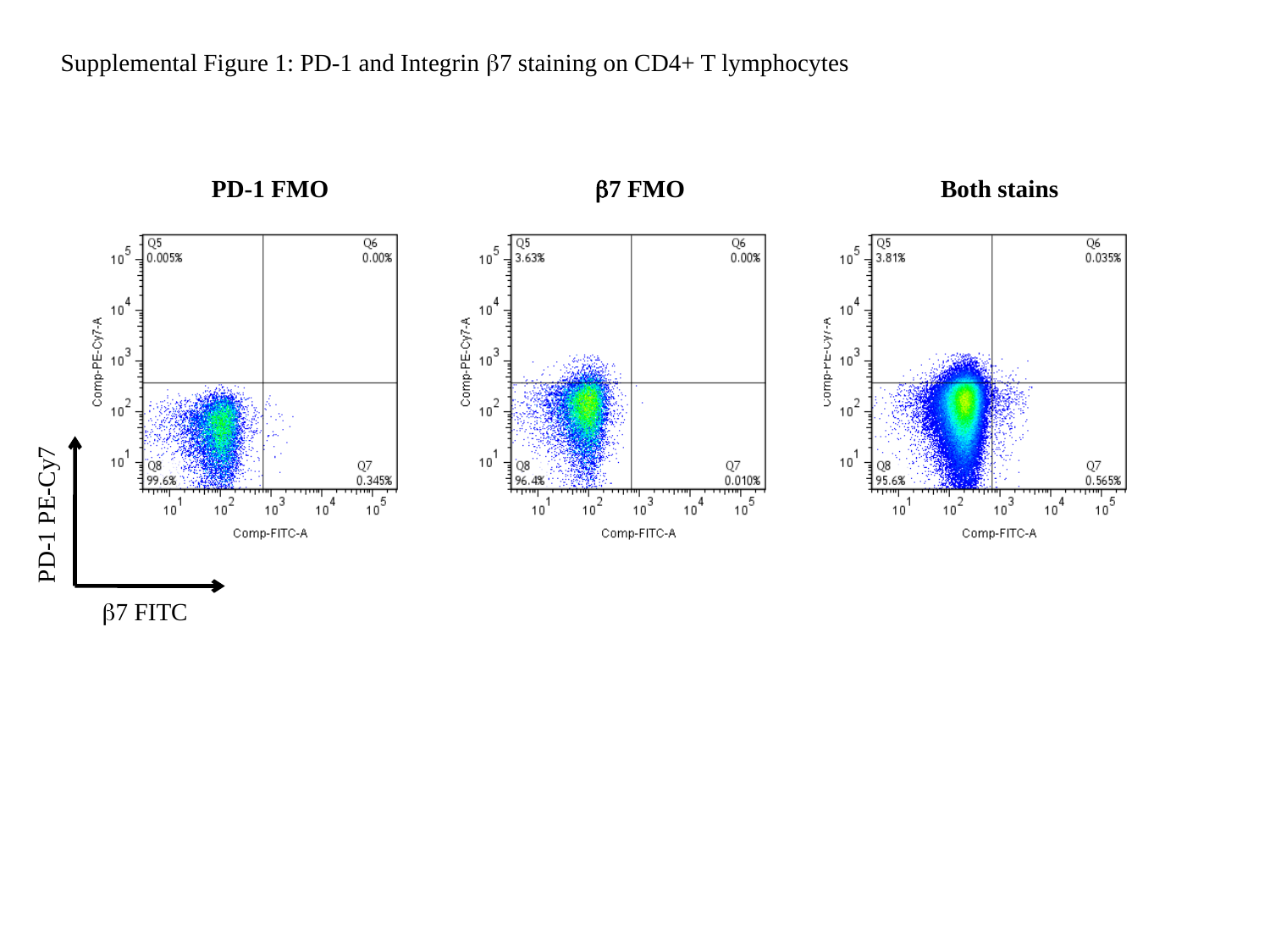

# Supplemental Figure 1: PD-1 and Integrin β7 staining on CD4+ T lymphocytes
PD-1 FMO
β7 FMO
Both stains
PD-1 PE-Cy7
β7 FITC
