# Supplementary figures and images for: Impact of antigen specificity on CD4+ T cell activation in chronic HIV-1 infection
Source: BMC Infect Dis. 2013 Feb 25;13:100. doi: 10.1186/1471-2334-13-100 (PMC3598342; doi:10.1186/1471-2334-13-100)

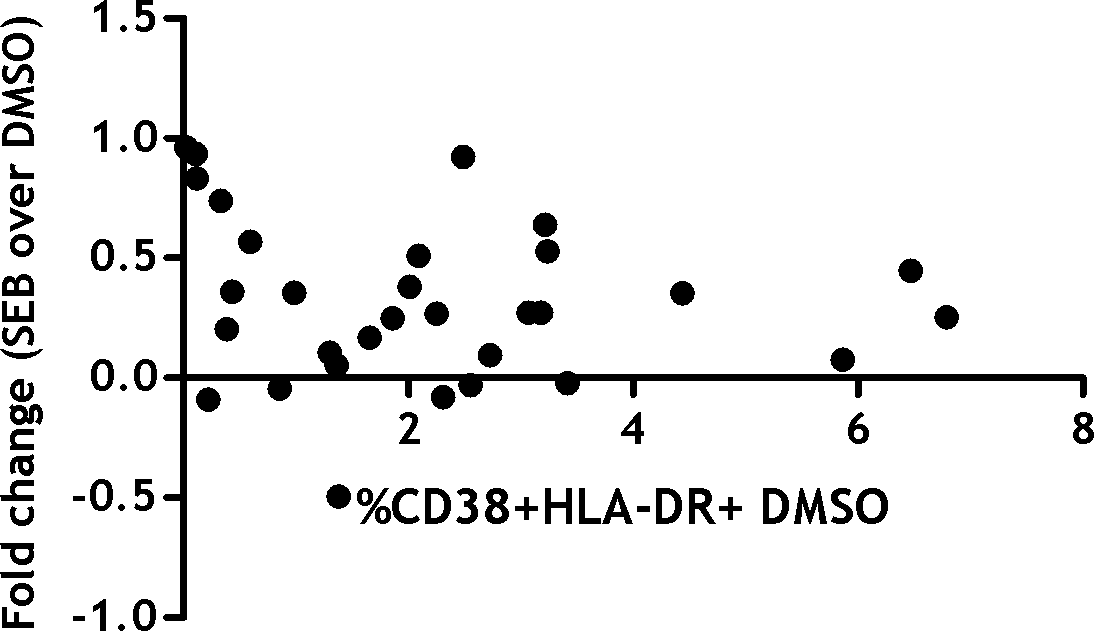

Supplement: Additional file 2: Figure S2 — Intracellular cytokine staining procedure has minimal effect on activation levels. The expression of CD38 and HLA-DR induced by the intracellular cytokine assay was assessed by comparing activation levels in the DMSO (negative) and SEB (positive) controls. Here the fold change demonstrated between DMSO and SEB is graphed against the percentage of activated CD4+ T cells in the DMSO control. There is no significant correlation (r = −0.18, p = 0.33). Donors with higher baseline levels of activation did not show any greater increase in activation through the assay than those with lower baseline levels of activation. Overall, the activation induced by the assay was no more than one times the background level. [file 1471-2334-13-100-S2.tiff]
